# Searching for the *Fusarium* spp. Which Are Responsible for Trichothecene Contamination in Oats Using Metataxonomy to Compare the Distribution of Toxigenic Species in Fields from Spain and the UK

**DOI:** 10.3390/toxins14090592

**Published:** 2022-08-28

**Authors:** Jéssica Gil-Serna, Belén Patiño, Carol Verheecke-Vaessen, Covadonga Vázquez, Ángel Medina

**Affiliations:** 1 Department of Genetics, Physiology and Microbiology, Faculty of Biology, University Complutense of Madrid, Jose Antonio Novais 12, 28040 Madrid, Spain; 2 Applied Mycology Group, Cranfield Soil and AgriFood Institute, Cranfield University, Cranfield MK43 0AL, UK

**Keywords:** metataxonomy, *Fusarium*, trichothecenes, T-2 toxin, HT-2 toxin, deoxynivalenol, soil

## Abstract

The contamination of oats with *Fusarium* toxins poses a high risk for food safety. Among them, trichothecenes are the most frequently reported in European oats, especially in northern countries. The environmental conditions related to the climate change scenario might favour a distribution shift in *Fusarium* species and the presence of these toxins in Southern European countries. In this paper, we present an ambitious work to determine the species responsible for trichothecene contamination in Spanish oats and to compare the results in the United Kingdom (UK) using a metataxonomic approach applied to both oat grains and soil samples collected from both countries. Regarding T-2 and HT-2 toxin producers, *F. langsethiae* was detected in 38% and 25% of the oat samples from the UK and Spain, respectively, and to the best of our knowledge, this is the first report of the detection of this fungus in oats from Spain. The relevant type B trichothecene producer, *F. poae*, was the most frequently detected *Fusarium* species in oats from both origins. Other important trichothecene producers, such as the *Fusarium tricinctum* species complex or *Fusarium cerealis*, were also frequently detected in oat fields. Many *Fusarium* toxins, including T-2 and HT-2 toxins, deoxynivalenol, or nivalenol, were detected in oat samples. The results obtained in this work revealed a clear change in the distribution of trichothecene producers and the necessity to establish the potential of these species to colonize oats and their ability to produce mycotoxins.

## 1. Introduction

*Fusarium* toxins are considered among the main mycotoxins in cereals due to their frequent occurrence and their harmful effects on animals and humans [1]. The maximum levels of many *Fusarium* toxins in cereals are regulated by the European Union, including trichothecenes, fumonisins, and zearalenone (ZEN) [2,3].

Among all cereals, the cultivation of oats stands out in the European Union since its production has been increasing every season. From 2019 to 2020, the harvested oats increased up to 22.1% probably due to the sharp rise in the cultivation area (7.2% higher) [4]. Although several toxins have been reported in this cereal, trichothecenes are considered the most important toxins in this matrix due to their toxic properties and their widespread distribution [5,6,7]. T-2 toxin and its deacetylated derivative HT-2 are the most toxic compounds included in this group, as they cause serious problems in the hematologic and immune systems [8]. Although it is currently an indicative level, the European Commission set a recommendation of 200 µg/kg in oats for direct human consumption [9]. Other *Fusarium* toxins regulated in oats include deoxynivalenol (DON) (1750 and 750 µg/kg for unprocessed oats and derived products intended for human consumption, respectively) and ZEN (100 µg/kg for unprocessed oat or 75 µg/kg if intended for direct human consumption) [2].

The presence of T-2 and HT-2 toxins has been demonstrated in many European countries of all latitudes and several food commodities although oats are the most frequently contaminated [10]. Several research papers have reported the presence of variable levels of type A trichothecenes in oat samples from Northern and Central Europe and related their distribution to weather conditions and agricultural practices [11,12,13,14]. The United Kingdom (UK) is currently considered the country with the highest type A trichothecene contamination levels [15].

Traditionally, species of the genus *Fusarium* have been considered the main producers of type A trichothecenes, and most of the research carried out to date has been focused on their study. T-2 and HT-2 toxin contamination in the UK has been attributed to *Fusarium langsethiae*, and a strong correlation was found between the presence of this fungus and toxin contamination in oats [16,17]. In other Central and Northern European countries, this fungus is also considered to be largely responsible for the presence of type A trichothecenes in oats, as is the case in Sweden [18,19], Norway [20], Switzerland [21], and Finland [22]. In some cases, the type A trichothecene producer *F. sporotrichioides* co-occurred with *F. langsethiae* but always in small quantities. The fact that *F. langsethiae* might predominate in colder climates of Northern Europe is confirmed by ecophysiological in vitro studies [23,24,25]. However, *F. sporotrichioides* seems to be better adapted to warmer temperatures and water stress, which may also explain its occurrence in Southern Europe [24,26].

Type B trichothecenes, mainly DON, have been also reported in oats from Northern European countries, and *F. graminearum* is usually described as the most important producing species [19,20,22,27,28]. This toxin has also been detected in oats from Southern Mediterranean countries although at low levels [29].

Climate change predictions reveal that *Fusarium* species are very likely to vary their distribution in Europe due not only to variations in ambient temperature and humidity but also to changes in crop management, which will have to be applied to adapt to the new conditions (earlier harvests, new crops, etc.) [30,31]. Fungi have an enormous capacity to adapt to changes, and different authors have highlighted that high resilience is very common in toxigenic fungal species [32]. Therefore, under the new conditions posed by climate change, it is expected that fungi can evolve quickly and adapt easily, leading to a prediction of an increase in the mycotoxin contamination of crops [11]. For example, in the case of type A trichothecene-producing species, some research has already detected the presence of *F. langsethiae* on wheat in Italy, confirming its adaptation and migration towards regions with warmer temperatures [33,34,35].

Knowing which species is/are responsible for producing mycotoxins is a key point to determine the best method to control their development and, therefore, avoid contamination by these toxins. In previous studies, the presence of T-2 and HT-2 toxins has been demonstrated in Spain in barley [36], maize [37], and oats [38], but it was not possible to determine the presence of potential producers of the genus *Fusarium* either by PCR-based molecular techniques or conventional isolation in culture. The isolation of *Fusarium* species, including *F. langsethiae*, might be complicated since they have a slow growth rate, and their colony morphology is lax; therefore, they can easily go unnoticed compared to other faster-growing fungi, and their isolation can be very problematic [39]. Moreover, there are many genera also capable of synthesizing trichothecenes, and some of them are very common in cereal crops such as *Cephalosporium*, *Microcyclospora*, *Myrothecium*, *Peltaster*, *Spicellum*, *Stachybotrys*, *Trichoderma*, and *Trichothecium* [40,41]. In this context, amplicon-based metagenomic approaches (metataxonomy) have been proposed as an alternative to traditional techniques to evaluate the whole complexity of fungal communities including underrepresented or unculturable species [42,43].

This paper presents an ambitious work to determine the species responsible for type A trichothecene contamination in Spain and to compare the results in a Northern European scenario. A metataxomic approach was applied to study the complete fungal diversity in oat fields (grain and soils) in Spain and the UK to (i) search for potential species capable of producing T-2 and HT-2 toxins, (ii) compare the risk for trichothecene contamination in oat fields from Southern and Northern Europe, and (iii) evaluate fungal diversity in soil and oat samples mainly focusing on mycotoxin-producing species. Moreover, the occurrence of certain fungal metabolites in oat samples, including mycotoxins, was also evaluated to establish the risk that oats can pose to food safety.

## 2. Results

### 2.1. Metataxonomic Study of Fungal Communities in Oat Fields

The mycobiota present in the oat field was determined through an amplicon-based metagenomic approach using the ITS2 region of the rDNA. Fungal taxonomy was studied both in oat grains and soil samples. Rarefaction analysis was performed to check if the sequencing depth was sufficient to observe the richness of the samples. In all cases, the curves reached the plateau, and they were close to saturation; therefore, the number of sequences obtained was enough (Supplementary Figure S1). In all samples, the Q20 reached at least 98.5%, whereas Q30 was higher than 94.5%, confirming the quality of the sequencing process.

#### 2.1.1. Diversity Analysis

**Figure 1:**
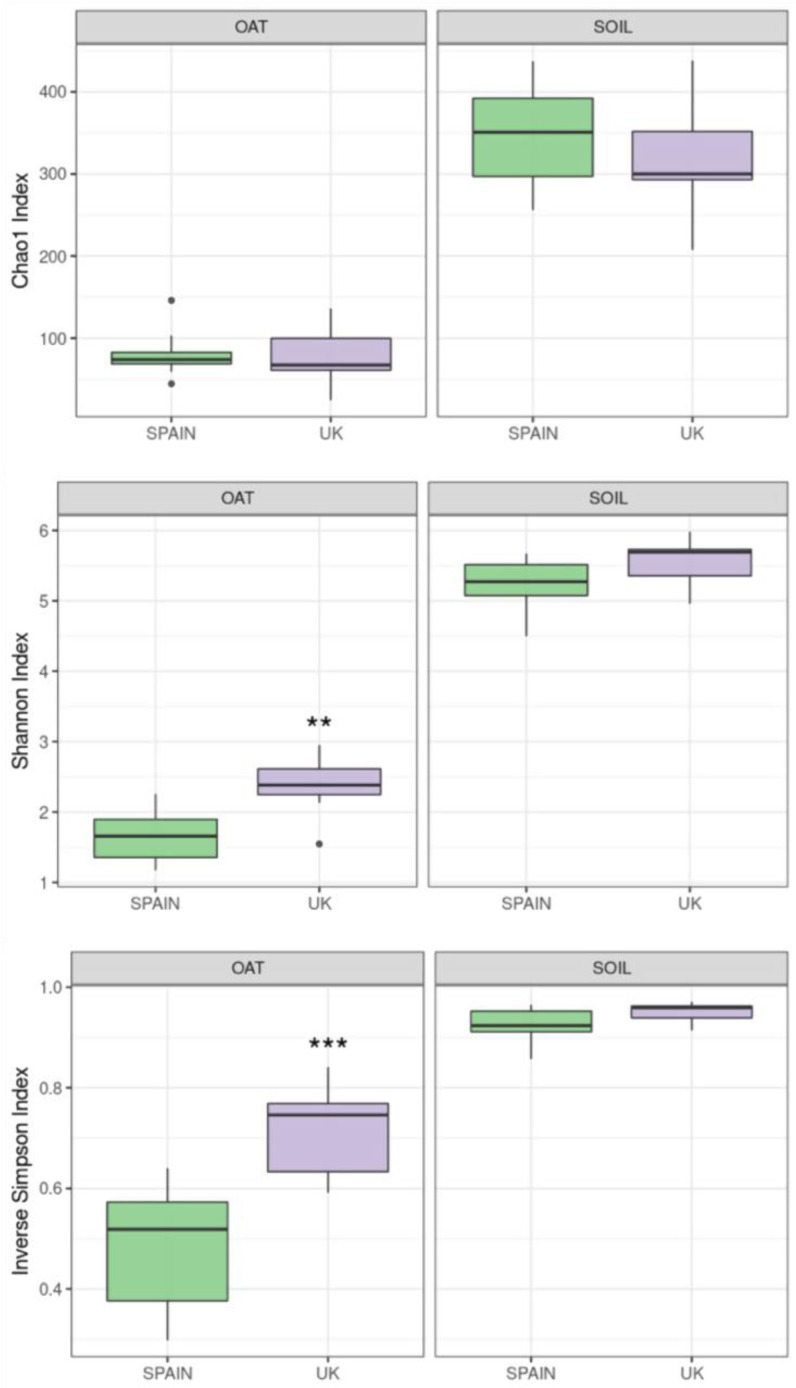
Alpha diversity indexes in oat grains and soil. The results include a richness index (chao1), an evenness index (Shannon index), and a dominance index (inverse Simpson). The stars above the bars indicate statistically significant differences between the origin of the samples (**: 0.001 < *p* < 0.01; ***: *p* < 0.001).

Figure 1 shows the results of alpha-diversity indexes comparing samples from both types and origins. Considering the three diversity indexes analysed, fungal communities from soil were rich and diverse, and presented a high species evenness. The high values of Chao1 and Shannon indexes are related to rich and uniform fungal communities observed in soil samples from oat fields, considering both the absolute and relative abundance of OTUs, respectively. The high values of the inverse Simpson index (close to 1) indicate that there is no species dominance in the soil of oat fields. No statistically significant differences were found regarding the origin of the samples in any case.

All alpha-diversity indexes were lower in the case of oat samples in comparison with soil samples. There are no differences in species richness regarding the origin of the samples, similar to the chao1 index, although the samples from Spain presented lower species evenness. Moreover, a clear species dominance was found in the case of Spanish oats with low values of the inverse Simpson index, which were significantly lower than those found in British samples.

**Figure 2:**
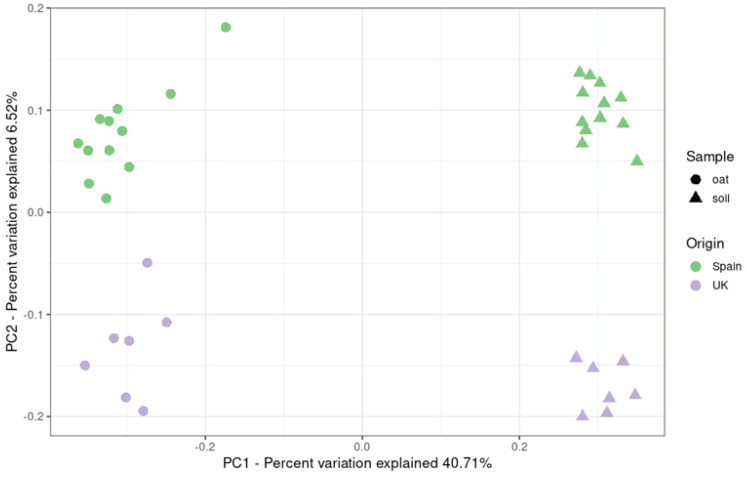
Principal coordinates analysis (PCoA) derived from unweighted UniFrac calculated from OTU relative abundance. Each dot represents a soil (triangle) and oat grain (circle) sample from Spain (green) or the UK (purple).

Beta diversity of the communities was also studied using a PCoA analysis (Figure 2). This unweighted UniFrac analysis is based on the presence of different taxa and revealed two clear principal coordinates that explained almost 50% of the variability observed. The OTU abundance of the fungal communities is related to the origin of the analysed variables and the type of sample.

#### 2.1.2. Taxonomical Assignments at the Genus Level

**Figure 3:**
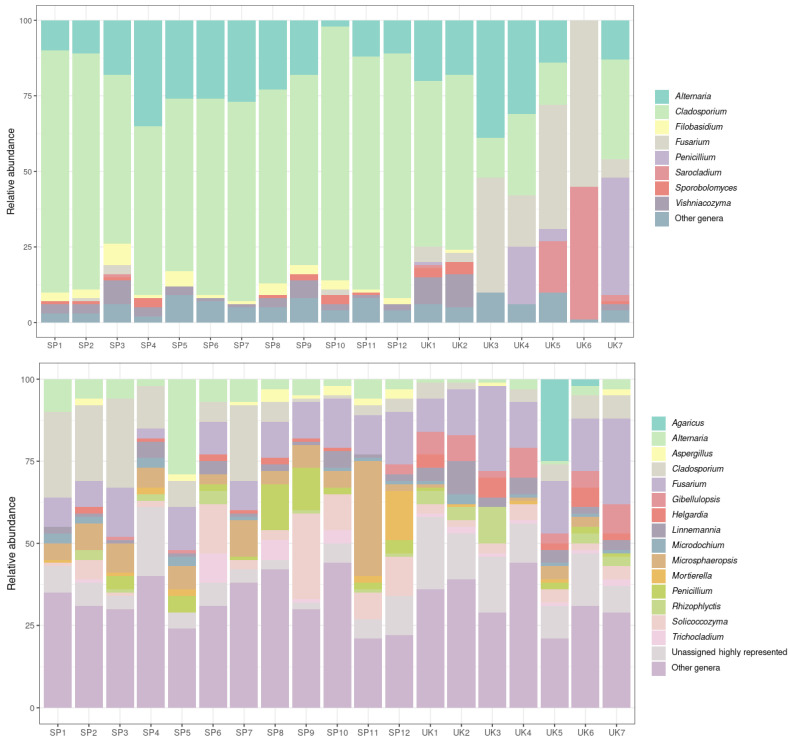
Histograms of the relative abundance of fungal genera in oat grains (**above**) and soil samples (**below**). Samples from SP1 to SP10 were collected in Spain and from UK1 to UK7 came from the UK.

The taxonomy of the OTUs found in the samples was analysed using the UNITE database for comparison. The histograms generated at the genus level with the soil and oat samples are shown in Figure 3. Regarding oat grains, *Alternaria* and *Cladosporium* were by far the most abundant genera in Spanish samples. The high percentage of *Cladosporium herbarum*, representing more than 80% of the total diversity in some oat samples, could be related to the low values of inverse Simpson index found in Spanish oats, indicating that it is a dominant species in samples from this origin. These two genera were also abundant in most of the samples from the UK. The most significant issue when the origin of the oat samples was compared was the high abundance of *Fusarium* species in most of the British oats analysed.

The histogram representing the abundance of fungal genera in soil samples is also included in Figure 3. In general, all the samples are more heterogeneous and present a high number of fungal genera. It is interesting to highlight that the *Cladosporium* genus is quite abundant in soil samples from Spain, but *Fusarium* is also highly represented. The *Fusarium* genus is also quite abundant in the soil of oat fields in the UK.

**Figure 4:**
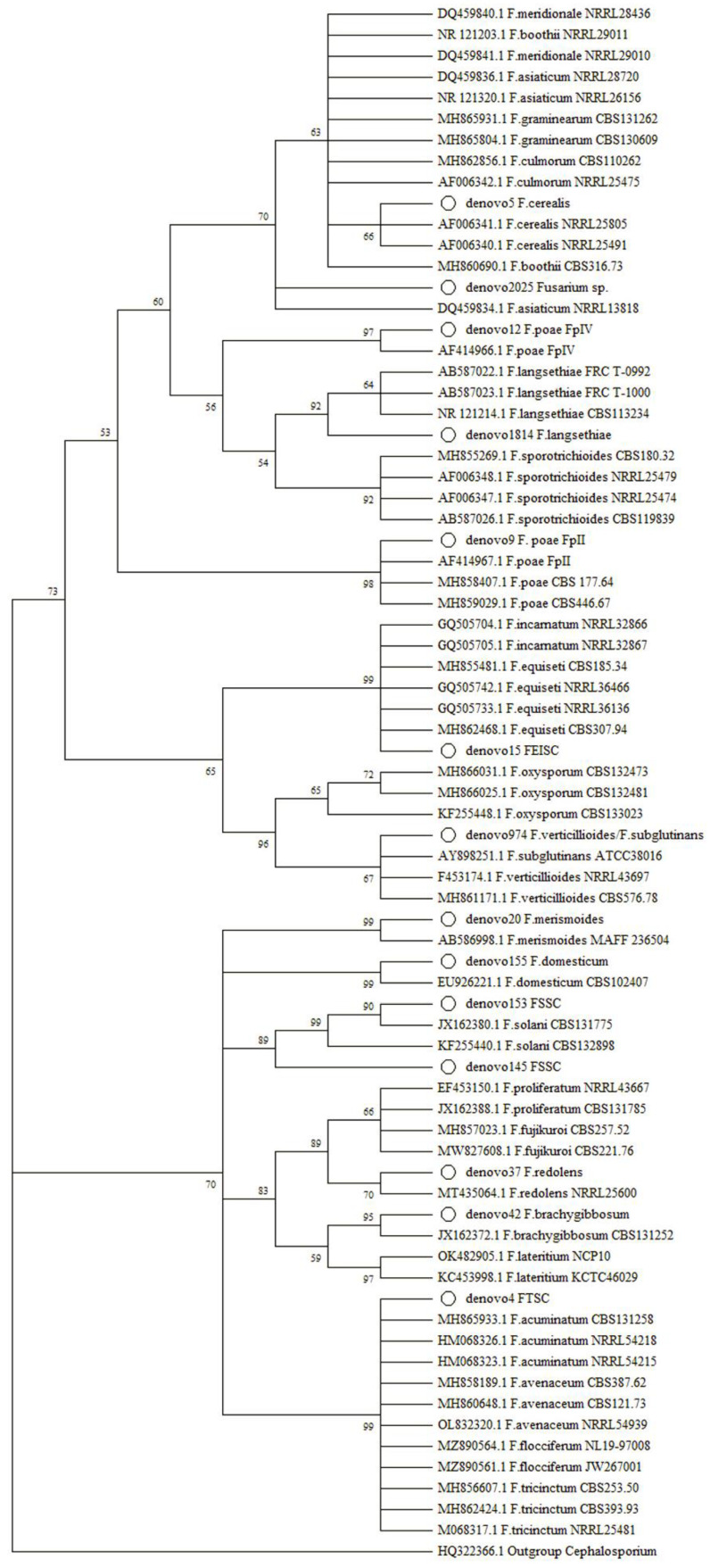
Maximum likelihood phylogenetic tree of the ITS2 sequences from *Fusarium* OTU obtained in this work together with several sequences retrieved from databases. The tree with the highest log likelihood (−1321.53) is shown. The taxa obtained in this work are marked with a white circle. The accession numbers of the sequences are indicated in the branches. Bootstrap values shown in the nodes and branches with values less than 50 were collapsed. FSSC, *Fusarium solani* species complex; FTSC, *Fusarium tricinctum* species complex; FEISC, *Fusarium equiseti-incarnatum* species complex.

One of the main focuses of this work was the study of trichothecene-producing species in oat samples, and the contamination with these toxins is usually related to the presence of *Fusarium* spp. To confirm the assignment of *Fusarium*, OTU was correctly performed, and a phylogenetic tree was constructed using the ITS2 region of the detected *Fusarium* spp. together with a complete dataset of sequences retrieved from databases. The phylogeny results are shown in Figure 4.

Most of the OTUs were successfully and unequivocally assigned to a *Fusarium* species after phylogenetic analysis, such as *F. cerealis*, *F. langsethiae*, *F. merismoides*, *F. redolens*, *F. brachygibbosum*, and *F. domesticum*. It is interesting to highlight that the ITS2 region allowed us not only to identify *F. poae* at the species level but also to discriminate among the subgroups, and both Fp II and Fp IV were detected in the samples.

In other cases, it was not possible to assign the reads to a species, such as in the case of the read denovo15, which was only assigned to the *F. equiseti-incarnatum* species complex, or the read denovo974, which might be related to *F. verticillioides* or *F. subglutinans*. It was not possible to finish the assignation at the species level of three reads, and they were assigned to the *F. tricinctum* species complex or the *F. solani* species complex. The less abundant *Fusarium* OTU detected in denovo2025 was unsuccessfully assigned to a species or group since the ITS2 region does not allow for discrimination among relatively distant species included in the *F. graminearum* and *F. culmorum* groups, and therefore, it is considered as *Fusarium* sp.

#### 2.1.3. Study on the Occurrence of *Fusarium* spp.

**Figure 5:**
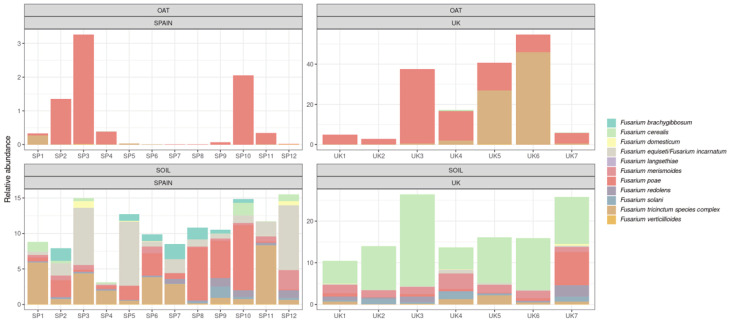
Histograms of the relative abundance of *Fusarium* spp. in oat grains (above) and soil samples (below). Samples from SP1 to SP10 were collected in Spain, and those from UK1 to UK7 came from the UK.

Once the correct taxonomical assignment of *Fusarium* reads was confirmed, the histogram with their distribution was studied (Figure 5), and the table with the abundance percentages of each *Fusarium* spp. is included in the Supplementary Materials (Table S2). Several trichothecene-producing *Fusarium* species were detected in oat samples. Regarding T-2 and HT-2 toxin producers, *F. langsethiae* was detected in 38% and 25% of the oat samples collected in the UK and Spain, respectively. To the best of our knowledge, this is the first report of the detection of *F. langsethiae* in oats from Spain. In the case of type B trichothecene producers, the most frequently found and the most prevalent in most of the samples was *F. poae*, which occurred in 80% of Spanish oats and all samples from the UK. This latter occurrence was quite significant since *F. poae* constitutes a high percentage of the total diversity; such is the case of UK3, which represents 37% of the total diversity. Both subgroups (Fp II and Fp IV) were present in samples from Spain and the UK. *Fusarium cerealis* is also a potential type B trichothecene producer and has been frequently detected in oats, mainly from the UK (six out of seven contaminated samples). This species was only detected in one Spanish grain sample.

Other potential producers of both A and B types of trichothecene might be the species of the *F. tricinctum* species complex. The read denovo4 was found in 67% and 100% of the oat grains from Spain and the UK, respectively. It is again in these latter samples in which higher percentages were obtained; for example, the OTU assigned to the *F. tricinctum* species complex represents up to 46% of the total diversity in the case of the UK6 sample. 

Potential fumonisin-producing species were also found in the Spanish and British samples (25 and 38% of contaminated samples, respectively), but it was not possible to determine if the OTU corresponded to *F. verticillioides* or *F. subglutinans*. 

**Figure 6:**
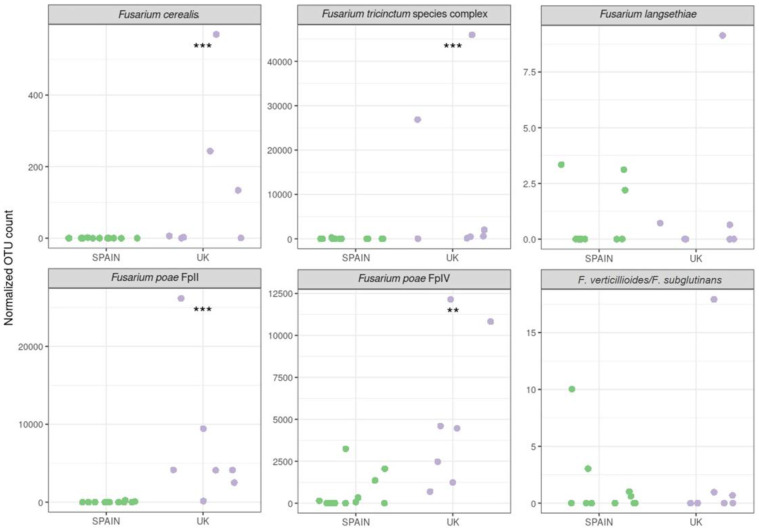
Comparison of the normalized OTU count for the main toxigenic *Fusarium* species detected in oat grains from Spain and the UK. The stars indicate statistically significant differences between the origin of the samples (**: 0.001 < *p* < 0.01; ***: *p* < 0.001).

The differences in the occurrence levels of the most relevant species regarding their potential to produce mycotoxins in oats were statistically evaluated (Figure 6). For this analysis, OTU counts were normalized using the library size to avoid misinterpretation due to the sequencing depth. In general, potential trichothecene-producing species occur more frequently in British oats. There is a statistically significant higher occurrence of the *F. tricinctum* species complex, *F. cerealis*, and the two groups of *F. poae* in oats from the UK compared from Spain. Therefore, this high occurrence might be related to the contamination of the samples with both type A and B trichothecenes.

In general, the soil samples from both origins present a relatively heterogeneous abundance of *Fusarium* at the species level. It is interesting to highlight that some species that are frequently found in soil samples are not able to appear in oat grains, such as *F. brachygibbosum* or the OTU assigned to the *F. equiseti-incarnatum* species complex. On the contrary, *F. langsethiae* was present only in oat samples from both origins, but it was not found in the soil (Figure 5 and Table S2).

#### 2.1.4. Study on the Occurrence of Other Potential Trichothecene-Producing Species

Considering the focus of this work was to determine the contamination source of type A trichothecene in oats, other species that have been reported as producers were also screened in the taxon analysis. Some potential producers such as *Myrothecium* sp., *Stachybotrys* spp., *Trichothecium* spp., and *Peltaster* sp. were detected in a few samples from the UK and Spain but only in the case of soil samples. *Trichoderma* spp. was detected in variable levels in oat fields from both origins, but it was not able to contaminate oats, and they were only detected in soil samples.

### 2.2. Contamination of Oat Samples with Mycotoxins

Five out of the fourteen screened mycotoxins were not detected in any oat samples, including relevant *Fusarium* toxins such as zearalenone, fumonisins B1 and B2, 15-ADON, or Fusarenon X. The results of the rest of the mycotoxins analysed in oat grains are shown in Table 1.

In general, oat samples from the UK are more contaminated by *Fusarium* toxins when compared to Spanish samples. In the case of Spanish oats, 58% of the samples were not contaminated by any *Fusarium* toxin; only one toxin was found in 25% of the grains, whereas one sample was contaminated by two *Fusarium* toxins (SP1). It is important to highlight the SP2 sample in which five *Fusarium* toxins were found, and it was the only Spanish sample contaminated by T-2 and HT-2 toxins. Moreover, the concentration found exceeds the levels proposed in the European Recommendation (1000 µg/kg for the sum of both toxins in unprocessed oat). The most frequently found toxin in Spanish oats was DON (5 out of the 12 samples) although in all cases, the contamination levels were lower than the maximum allowed levels in oats regarding the European regulation (1200 µg/kg).

All British oats were contaminated by at least two of the analysed *Fusarium* toxins. In the UK3 sample, the co-occurrence of five mycotoxins was detected. Among type A trichothecene, the compounds detected at higher levels were the T-2 toxin, which was present in two samples, as well as diacetoxyscirpenol (DAS), which was also detected in two oat samples. Regarding type B trichothecenes, nivalenol (NIV) was the most frequent and was present in 57% of the British oat samples, reaching high levels in some cases, for example, in UK1. Beauvericin was also one of the most relevant toxins found in oats from the UK, with 86% of contaminated samples with levels up to 1266 µg/kg.

## 3. Discussion

The *Fusarium* genus is one of the most important fungi occurring in cereals because they can be both phytopathogenic and toxigenic, and they are always the focus of prevention strategies [1]. Among *Fusarium* toxins, trichothecenes are one of the most toxic groups, and cereals are their primary source in human and animal diets [5,7]. The general cereal production in the European Union has slightly decreased in recent years although an important rise in oat production is occurring [4]. Oats are now considered a trendy product, and the demand for oat-based products and forecasts indicate that its market is expected to drastically increase worldwide [44]. In this regard, it is essential to guarantee consumers’ safety, and the presence of mycotoxins should be strictly controlled in both raw and processed oats [45]. Moreover, the co-occurrence of several *Fusarium* mycotoxins in oats has been commonly reported, which might pose a high risk for food safety [12,46,47].

The presence of T-2 and HT-2 toxins in oats in Northern Europe has been extensively studied [16,17,18,19,20,21,22]. However, the potential producers of these toxins have not yet been elucidated in Southern European countries. It is important to consider that Spain is the second largest producer of oats in the European Union, and its production in the period 2019–2020 increased by 64% [4]. Thus, it is essential to determine which fungi might be involved in the contamination of type A trichothecene in this country. 

It is widely known that *F. langsethiae* is one of the most important T-2- and HT-2-producing species in oats in northern latitudes [5]. However, their presence in oats from more temperate countries has not yet been reported, and it has only been demonstrated in barley and wheat from Southern European countries [33,34,35]. On the other hand, it is also known that infection levels of the fungus are hardly detected and only in special cases produce visible symptoms in oat plants; therefore, some authors maintain the opinion that this fungus is an endophyte rather than a pathogen [48]. This symptomless infection of oats allows the fungus to remain unnoticed, enabling the accumulation of high levels of mycotoxins in the grains. The low levels of fungal inoculum reached in the grains together with its slow growth rate might be related to the difficulty of its isolation and detection [39].

Metataxonomic approaches are considered a good strategy to detect microorganisms that are rare and present a low abundance, including fungal pathogens [49]. Using this metabarcoding analysis, we detected low levels of *F. langsethiae*, and to the best of our knowledge, this is the first report on the occurrence of this fungus in Spanish oats. It is interesting to note that only one Spanish sample was contaminated by T-2 and HT-2 toxins, and it was one out of the three samples on which *F. langsethiae* was detected. Therefore, this could be an indicator that *F. langsethiae* might also be contributing to some extent to the T-2 and HT-2 content in oats produced in South European countries. *Fusarium langsethiae* was also found in oats from the UK although the occurrence of this kind of type A trichothecenes was not very high. Although high levels of T-2 and HT-2 toxins are often detected in British oats, they are known to be highly dependent on the weather conditions of the season [13].

In all cases, *F. langsethiae* was detected in grains, and the fungus was never found in soil samples from any of the origins. The infection route of *F. langsethiae* remains unclear because it is difficult to understand the initial source of the inoculum [48,50]. This fungus is not able to produce chlamydospores, which makes its survival in the soil difficult [51], and the results obtained in our work confirm the lack of inoculum in the kind of samples. This fact opens the field for new studies to determine the ecological cycle of *F. langsethiae*, which seems to be different from other *Fusarium* species.

Some ecophysiological studies predict a higher occurrence of *F. sporotrichioides* in southern regions of Europe because this fungus is more adapted to warmer regions than other type A trichothecene producers [24,26]. However, in our work, *F. sporotrichioides* was not detected in any sample from any origin. In recent years, different studies performed on other cereals from Spain have revealed a clear decrease in *F. sporotrichioides* occurrence [37,52], which may indicate a change in its global distribution. 

The ability to produce trichothecenes by *F. poae* has been extensively studied, and different strains have been reported to produce a variety of *Fusarium* toxins, including type A (T-2 and HT-2 toxins, DAS, T-2 treaol, NEO, etc.) and type B trichothecenes (DON, and NIV), as well as beauvericins [53,54,55]. Although *F. poae* is one of the most frequently detected species in many studies regarding oats in Europe [19,20,21,56] and Canada [57,58], the relation between the occurrence of this fungus and high levels of any mycotoxin has not been established. Some authors consider that the attribution of production of some mycotoxins such as T-2 toxin to *F. poae* is likely to be a misidentification of the strains that belong to *F. sporotrichioides* or *F. langsethiae* [59]. Edwards et al. [16] found a weak correlation between contamination levels and NIV content, whereas Islam et al. [58] related the occurrence of *F. poae* with high levels of NIV and beauvericins. This might be the case of the results obtained in this work since high levels of contamination of *F. poae* were found in oats from the UK, and the most frequently occurring mycotoxins are NIV and beauvericins.

Vanheule et al. [55] characterized different chemotypes in *F. poae*, which can synthesize different kinds of trichothecenes. However, the genetic variability in gene clusters could not explain variations in the chemotype. The knowledge of the genetic basis of mycotoxin production is essential to establish the ability of the species to produce trichothecenes, and therefore, extensive work might be needed to elucidate it. The ITS2 region used for taxonomic assignment in our work allowed us to discriminate among the *F. poae* clonal populations described elsewhere and that have been related to different geographical locations [60]. However, our results indicate that both groups (II and IV) independently detected the origin of the samples, and both of them occurred in the oat grains and the soil.

The *F. tricinctum* species complex gathers eleven species that are usually associated with the development of *Fusarium* head blight of cereals as well as the mycotoxin contamination of the grains [6]. This group includes trichothecene-producing species, such as *F. acuminatum* and *F. armenicum*; however, the reports of trichothecene production by *F. avenaceum* or *F. tricinctum* are probably incorrect and due to misidentification [59]. Nevertheless, in this work, the ITS2 region does not allow for discrimination among the closely related species in this complex, and considering their different toxigenic potential, it is not possible to establish their contribution to the total amount of mycotoxins detected in the samples. The lack of discrimination of the ITS2 region found in some groups is by far the most significant limitation of the metataxonomic approach used. In this regard, it would be more interesting to apply a different metabarcoding target, such as elongation factor 1-α, which provides higher levels of polymorphism to discriminate among species [61].

*Fusarium graminearum* has long been considered the main source of DON in European countries, and a strong correlation was found between DON levels and fungal contamination in oats [19,20,22,27,28]. In our work, the presence of this fungus was not found in oat fields either in the grains or in the soil. However, high DON levels were detected in some oat samples; therefore, a shift in *Fusarium* species might be occurring, and the *F. graminearum* niche might be occupied by other potential DON producers. *Fusarium cerealis* is morphologically very similar to *F. graminearum* and *F. culmorum*, and they are indistinguishable using conventional identification methods [59]. Palacios et al. [62] revealed the ability of *F. cerealis* not only to produce NIV, but some isolates also synthesize high levels of DON. Almost all oat grains taken from fields in the UK presented high levels of NIV and were contaminated by *F. cerealis*, which might be contributing to the level of this toxin in them. However, it was only present in one Spanish sample and represents a very low percentage of the total diversity.

DON was the most frequently detected toxin in Spanish oats although in all cases, the levels exceeded the legal regulation [2]. Some studies have detected this toxin in oats or oat-based products in Southern Europe, but these works have not been accompanied by the study of fungal diversity in the samples [29,38,63]. However, in our work, even using the highly sensitive amplicon-based metagenomics approach, we were not able to determine which species might be the potential producer of DON in Spanish oats.

Although in this work, fumonisins were not detected in oats in any case, potential fumonisin-producing species were detected in 33 and 43% of grain samples from Spain and the UK, respectively. In a recently published work, Tarazona et al. [38] detected fumonisin B1 and B2 in oats collected in Spain. Other recent studies demonstrated the occasional occurrence of fumonisins in oats and oat-based products worldwide [64,65,66]. Therefore, although they do not seem to pose a substantial risk, due to the presence of potential producers and an increasing number of studies reporting fumonisins in oats, the presence of these toxins should be carefully monitored. 

The primary source of *Fusarium* inoculum for cereal contamination is the soil where they arrived from crop residues [50,67]. Many *Fusarium* species are capable of resistance in the soil, and they can infect the plant the next season after their dispersion by wind or rain [68]. The soil is a complex ecosystem, and the microorganisms living in there establish complex ecological interactions that may affect the survival of other species, including mycotoxigenic species [69]. In a recently published work, Cobo-Díaz et al. [70] demonstrated the relationship between the composition of bacterial and fungal communities and *Fusarium* spp. occurrence in maize soils. On the other hand, Xiong et al. [71] observed that the higher fungal diversity of soils might be related to a suppression of *Fusarium* wilt disease. However, in our work, we found no evidence that the diversity of fungal communities might be affecting the occurrence of *Fusarium* spp. Although soil samples from British fields usually present higher percentages of *Fusarium* spp., no differences were found among the diversity indexes in both locations; therefore, neither the richness nor the evenness of soil fungal communities seems to be related to *Fusarium* abundance.

In this work, some important epidemiological conclusions might be addressed regarding the ability of the *F. equiseti-incarnatum* species complex to infect oats. This complex has been described recently, and little is known about its pathogenicity and mycotoxin potential [72,73]. In our work, we demonstrated the presence of this OTU in all soil samples independently of their origin. The species included in this group can produce resistance structures and are known to resist long periods in the soil, but our results indicate that they are not easily transferred from the soil to the grains since they were only detected in 28% and 17% of the British and Spanish grains, respectively, and always represented a relatively low abundance (less than 0.005%). Although this group is described as an important mycotoxin producer [72,74,75], its contribution to the mycotoxin content in oats should be carefully studied since it seems it is not capable of effectively colonizing the plants. A similar situation was found in the case of the endophytic fungus *F. redolens*, which was widespread in soil samples from both origins, but it seems not to easily reach oat grains. This species has been described as a beauvericin producer in other cereals, such as maize [76], but its contribution to mycotoxin content in oats should be carefully studied.

Finally, it is important to mention the presence of *F. brachygibbosum* in all Spanish fields analysed, mainly in the soil. This species was not detected in the UK. In a recently published study, the EFSA considered it as an important plant pathogen and recommended regarding it as a potential quarantine pest [77]. Moreover, the EFSA states that it is necessary to clarify its distribution and its potential impact. In our work, we demonstrated its presence in Spanish oat fields. Interestingly, in a recently published paper, Rabaaoui et al. [78] described that some isolates of *F. brachygibbosum* may have the ability to produce mycotoxins, including trichothecenes and beauvericin. Therefore, it seems necessary to perform new studies to determine the distribution and mycotoxin production potential of this fungal species. This will provide the necessary datasets to develop a risk assessment to evaluate the risk this species poses to agriculture and food safety.

## 4. Conclusions

The novel metataxonomic approach used in this work allowed to stablish the composition of the *Fusarium* mycobiota in oat fields in the UK and Spain and revealed the presence of species that had not been detected before. The results obtained in this work confirm a clear change in the distribution of *Fusarium* species, as is the case of the lack of *F. graminearum*, or the appearance of *F. langsethiae* in oats from Southern Europe. It is well-known that, under the forecasted environmental conditions due to climate change, different scenarios predict a shift in *Fusarium* species across Europe, which might affect the mycotoxigenic profile [30,31]. Considering the increasing importance of trichothecenes regarding human and animal health and the changes in the distribution of their producers, it is essential to establish the potential of these species to survive and colonise crops under these new environmental conditions and whether there will be any changes to their ability to produce mycotoxins. The information available in the literature is scarce and, in many cases, based on obsolete protocols for the identification of fungi, which led to misclassification of the isolates. In recent years, the taxonomy of the *Fusarium* genus has been constantly evolving and revised; nonetheless, it is essential to perform extensive studies to clarify which mycotoxins can produce each species to be able to properly evaluate their presence and, subsequently, predict the risk of mycotoxin contamination. Considering all the aspects discussed in this paper, it is clear that an integrated approach is required to reduce the occurrence of *Fusarium* species and therefore effectively prevent mycotoxin accumulation in the grains.

## 5. Materials and Methods

### 5.1. Sample Collection

Samples were collected in oat fields from Spain and the UK during summer 2020, two weeks before harvest. For each field, oat samples were collected at random, taking one sample every 3 m along the plot, reaching approximately 1 kg. Upon arrival at the laboratory, oat plants were threshed and milled using an IKA A11 Basic Mill (IKA, Staufen, Germany). Soil samples were also taken in the same fields following the same random approach mentioned above. In each step, approximately 300 mL of subsurface soil was taken, reaching approximately 3 L of soil in total. Once in the laboratory, the samples were dried at room temperature for 36 h and passed through a 200 µm pore-size sieve. All the processed samples were stored at 4 °C until analysis.

The 12 sample-collection points in Spain were selected in important oat-producing regions, such as Castilla y León, Madrid, and Castilla La Mancha (province of Toledo). In the case of the UK, all seven samples were collected in the Bedfordshire region. The exact coordinates of the sampling points are included in the Supplementary Materials (Table S1).

### 5.2. DNA Isolation and Metataxonomic Analysis

Genomic DNA extraction from oat flour was performed using the DNeasy Plant Mini Kit (QIAgen, Düsseldorf, Germany) starting from 100 mg of material, following the manufacturers’ instructions. Additionally, DNA isolation from soil samples was conducted using the DNeasy Power Soil Kit (QIAgen, Düsseldorf, Germany) starting from 250 mg powdered soil. In both cases, three DNA extractions were carried out for each sample and were subsequently mixed and concentrated using a vacuum concentrator (Concentrator plus, Eppendorf, Hamburg, Germany).

For the amplicon-based metagenomic analysis of the fungal communities, the ITS2 region of the ribosomal DNA was amplified using the ITS3/ITS4 universal primer pair [79]. Amplicon libraries were prepared using the Herculase II Fusion DNA Polymerase Nextera XT Index Kit V2 (Illumina, Foster City, CA, USA) and sequenced on MiSeq Illumina Platform in Macrogen facilities (Macrogen Inc., Seoul, Korea).

Data processing and analysis were performed using different bioinformatics software. The preliminary processing of the data was performed using FLASH 1.2.11 (Baltimore, MD, USA) [80] to merge paired-end reads. Subsequently, the demultiplexed sequences were filtered by quality, trimmed, and finally clustered into operational taxonomical units (OTU) using CD-HIT-OTU 4.5.5 (San Diego, CA, USA) [81]. Taxonomy assignments were carried out using the UNITE database [82], and further analyses on the alpha and beta diversity of fungal communities were performed using QIIME v. 1.8 (Boulder, CO, USA) [83].

### 5.3. Phylogenetic Analysis

A phylogenetic tree was inferred by using the maximum likelihood method in MEGA X [84] to confirm the correct classification of the *Fusarium* OTUs obtained after comparison with the databases. The sequences of the ITS2 regions corresponding to *Fusarium* spp. obtained in the metataxonomic analysis were combined in a dataset with other sequences retrieved from the NCBI nucleotide database. The dataset was composed of 75 nucleotide sequences, and a total of 259 positions were used for phylogenetic inference. Initial trees for the heuristic search were obtained automatically using the Neighbor-Join and BioNJ algorithms to a matrix of pairwise distances estimated using the maximum composite likelihood approach. A Kimura 2-parameter model with a discrete Gamma distribution was used to study the evolutionary rate differences among sites (shape parameter = 0.7282). The rate variation model includes some evolutionary invariable sites (47.53% sites). All positions containing gaps and missing data were eliminated.

### 5.4. Fungal Metabolite Determination in Oat Samples

The mycotoxin contamination of oat samples was performed following Isidro-Sanchez et al. [85] with slight modifications. Ultra-high-performance liquid chromatography–tandem mass spectrometry (UHPLC-MS/MS) was performed to measure mycotoxin concentrations, and the presence of 14 toxins was evaluated (T-2 and HT-2 toxins, 3-acetyldeoxynivalenol, 15-acetyldeoxynivalenol, nivalenol, deoxynivalenol, zearalenone, Fusarenon X, diacetoxyscirpenol, neosolaniol, 15-acetoxyscirpenol, beauvericin, and Fumonisins B1 and B2). Briefly, 100 mg milled oat samples were extracted with 500 µL acetonitrile/water/formic acid (79:20.9:0.1, *v*/*v*/*v*). Measurements were performed using a qTRAP-LC-MS/MS 6500+ system (Exion Serie). An ACE 3-C_18_ column (2.1 × 100 mm, 3 µm particle size; Hichrom, Berkshire, UK) equipped with a C_18_ security guard cartridge (4 × 3 mm, Gemini Agilent, Oxford, UK) kept at 60 °C was used for chromatographic separation with the following gradients: Solvent A: water:methanol:acetic acid (*v*:*v*:*v*, 89:10:1) and Solvent B: methanol:water:acetic acid (*v*:*v*:*v*, 97:2:1), both supplemented with 5 mM ammonium acetate, which was applied for 20 min, as defined in Malachová et al. [86]. The flow rate of the mobile phase was 0.6 mL/min, and the injection volume was set to 1 µL. MS/MS was performed in a scheduled multiple reaction monitoring (MRM) with a 60 s window. Analyst version 1.6.3 (AB SCIEX™, Framingham, MA, USA) and MultiQuant version 3.0.3 (AB SCIEX™, Framingham, MA, USA) were used for data acquisition and analysis, respectively.

### 5.5. Statistical Analysis

The non-parametric Wilcoxon test was applied to evaluate statistical significance regarding community diversity using R (version 4.1) (Auckland, New Zealand). Additionally, the occurrence of the most relevant fungal species regarding their toxigenic potential found in oat samples was also evaluated statistically to determine differences in their contamination levels related to geographical origin. The data did not fit the required normality criteria, and therefore, the non-parametric Wilcoxon test was also applied.

## Figures and Tables

**Table 1:** Concentration of each mycotoxin analysed in the corresponding oat sample in ng/g.

Sample	Origin	T-2	HT-2	3-ADON	DON	NIV	DAS	NEO	BEA	15-AS
SP1	Spain	ND	ND	ND	61.21	ND	ND	ND	ND	6.25
SP2	Spain	2123.58	7.5	423.45	73.1	ND	ND	ND	ND	4.07
SP3	Spain	ND	ND	ND	ND	ND	ND	ND	ND	ND
SP4	Spain	ND	ND	ND	ND	ND	ND	ND	ND	ND
SP5	Spain	ND	ND	ND	14.77	ND	ND	ND	ND	ND
SP6	Spain	ND	ND	ND	ND	ND	ND	ND	ND	ND
SP7	Spain	ND	ND	ND	ND	ND	ND	ND	ND	ND
SP8	Spain	ND	ND	ND	ND	ND	ND	ND	ND	ND
SP9	Spain	ND	ND	ND	ND	ND	ND	ND	ND	ND
SP10	Spain	ND	ND	ND	15.37	ND	ND	ND	ND	ND
SP11	Spain	ND	ND	ND	ND	ND	ND	ND	ND	ND
SP12	Spain	ND	ND	ND	10.79	ND	ND	ND	ND	ND
UK1	UK	ND	ND	ND	ND	2276.19	15.29	ND	98.95	86.60
UK2	UK	ND	ND	ND	63.42	ND	ND	ND	10.43	ND
UK3	UK	ND	ND	<LOQ	ND	424.65	0.69	ND	1266.00	54.75
UK4	UK	1400.23	ND	ND	ND	ND	ND	ND	44.03	ND
UK5	UK	ND	ND	<LOQ	ND	689.26	ND	ND	2.44	ND
UK6	UK	ND	ND	<LOQ	ND	ND	ND	5.29	ND	ND
UK7	UK	391.84	ND	ND	ND	233.27	ND	ND	456.30	5.23

DON, deoxynivalenol; 3-ADON, 3-acetylDON; NIV, nivalenol; DAS, diacetoxyscirpenol; NEO, neosolaniol; BEA, beauvericin; 15-AS, 15-acetoxyscirpenol; LOQ, limit of quantification; LOD, limit of detection; <LOQ, LOD < X < LOQ; ND, not detected.

## Data Availability

Raw data are available upon request; please contact the corresponding author.

## Supplementary Materials

The following supporting information can be downloaded at: https://www.mdpi.com/article/10.3390/toxins14090592/s1: Figure S1: rarefaction curves; Table S1: sample locations; Table S2: relative abundance of *Fusarium* spp. in oats.
