# Supplementary material for: Searching for the Fusarium spp. Which Are Responsible for Trichothecene Contamination in Oats Using Metataxonomy to Compare the Distribution of Toxigenic Species in Fields from Spain and the UK
Source: Toxins (Basel). 2022 Aug 28;14(9):592. doi: 10.3390/toxins14090592 (PMC9506359; doi:10.3390/toxins14090592)
Supplement: Supplementary file 1 [file toxins-14-00592-s001.zip › toxins-1853589-supplementary.pdf]

Supplementary Materials: Searching for the *Fusarium* spp. Which Are Responsible for Trichothecene Contamination in Oats Using Metataxonomy to Compare the Distribution of Toxigenic Species in Fields from Spain and the UK

Jéssica Gil-Serna, Belén Patiño, Carol Verheecke-Vaessen, Covadonga Vázquez and Ángel Medina

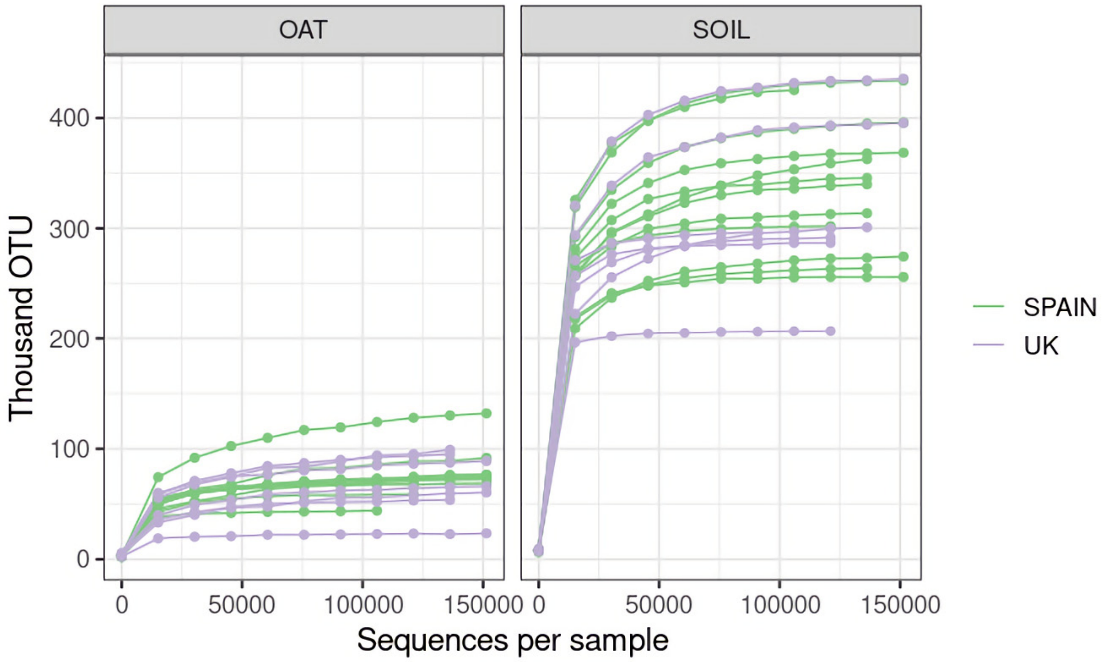

Figure S1. Rarefaction curves generated plotting the OTU number against the sequences obtained on each sample.

Table S1. Location of the oat fields where the samples were taken.

Code	Country	Location	Region	GPS coordinates
SP1	Spain	Santervás de Campos	Valladolid	42º13'47.7"N 5º1'5.3"W
SP2	Spain	Cervatos de la Cueza	Palencia	42º17'12.6"N 4º45'48.5"W
SP3	Spain	Villaconancio	Palencia	41°51'54.3"N 4°12'54.5"W
SP4	Spain	Mayorga	Valladolid	42°10'06.0"N 5°14'52.0"W
SP5	Spain	El Pardo	Madrid	40°31'53.2"N 3°46'54.4"W
SP6	Spain	Guismondo	Toledo	40°07'51.3"N 4°18'10.7"W
SP7	Spain	Santa Cruz del Retamar	Toledo	40°08'56.5"N 4°17'14.7"W
SP8	Spain	Nava de Sotrobal	Salamanca	40°52'14.1"N 5°14'38.6"W
SP9	Spain	Tordillos	Salamanca	40°52'26.4"N 5°14'40.7"W
SP10	Spain	Macotera	Salamanca	40°49'10.6"N 5°19'2.6"W
SP11	Spain	Villatoquite	Palencia	42°12'05.0"N 4°46'32.7"W
SP12	Spain	Renedo de Esgueva	Valladolid	41°39'37.7"N 4°37'55.4"W

UK1	UK	Turvey	Bedford	52°10'15.2"N 0°37'19.6"W
UK2	UK	Turvey	Bedford	52°10'42.8"N 0°37'20.1"W
UK3	UK	Carlton and Chellington	Bedford	52°11'20.3"N 0°34'37.4"W
UK4	UK	Thurleigh	Bedford	52°12'49.8"N 0°26'19.8"W
UK5	UK	Bolnhurst and Keysoe	Bedford	52°13'00.8"N 0°25'17.3"W
UK6	UK	Riseley	Bedford	52°15'40.5"N 0°27'56.2"W
UK7	UK	Riseley	Bedford	52°14'58.7"N 0°29'16.2"W

Table S2. Relative abundance of *Fusarium* spp. found in oat grain samples.

Sample	Origin	*F. brachygibbosum*		*F. cerealis*		*F. domesticum*		FEISC	
		SOIL	GRAIN	SOIL	GRAIN	SOIL	GRAIN	SOIL	GRAIN
SP1	Spain	ND	ND	1.320%	ND	0.025%	ND	0.455%	ND
SP2	Spain	1.776%	ND	0.370%	ND	ND	0.001%	1.707%	ND
SP3	Spain	ND	ND	0.428%	ND	0.942%	ND	8.084%	ND
SP4	Spain	0.001%	ND	0.159%	0.002%	ND	ND	0.153%	0.005%
SP5	Spain	0.961%	ND	0.021%	ND	0.072%	0.001%	9.045%	ND
SP6	Spain	0.952%	ND	0.003%	ND	0.003%	ND	0.764%	ND
SP7	Spain	2.130%	ND	ND	ND	ND	ND	1.967%	ND
SP8	Spain	1.700%	ND	0.005%	ND	ND	ND	0.996%	ND
SP9	Spain	0.521%	ND	0.066%	ND	ND	ND	0.627%	ND
SP10	Spain	0.526%	ND	1.787%	ND	ND	ND	1.070%	ND
SP11	Spain	0.032%	ND	0.022%	ND	ND	ND	2.089%	ND
SP12	Spain	ND	0.001%	0.938%	ND	0.584%	ND	9.114%	0.002%
UK1	UK	ND	ND	5.419%	0.134%	0.054%	ND	0.224%	ND
UK2	UK	ND	ND	10.478%	0.001%	0.007%	ND	0.139%	0.004%
UK3	UK	ND	ND	22.031%	0.006%	0.015%	ND	0.173%	ND
UK4	UK	ND	ND	5.286%	0.570%	0.082%	ND	0.966%	0.002%
UK5	UK	ND	ND	10.991%	0.003%	ND	ND	0.467%	ND
UK6	UK	ND	ND	12.388%	ND	ND	ND	0.235%	ND
UK7	UK	ND	ND	11.294%	0.243%	0.395%	ND	0.301%	ND

Sample	Origin	*F. langsethiae*		*F. merismoides*		*F. poae* II		*F. poae* IV	
		SOIL	GRAIN	SOIL	GRAIN	SOIL	GRAIN	SOIL	GRAIN
**SP1**	Spain	ND	ND	0.381%	ND	0.500%	ND	0.020%	0.070%
SP2	Spain	ND	0.002%	0.701%	ND	2.321%	ND	ND	1.351%
SP3	Spain	ND	0.003%	0.636%	ND	0.268%	ND	0.012%	3.239%
SP4	Spain	ND	ND	0.210%	ND	0.375%	0.236%	ND	0.141%
SP5	Spain	ND	ND	0.063%	ND	1.942%	ND	ND	ND
SP6	Spain	ND	ND	0.922%	ND	3.156%	ND	ND	ND
SP7	Spain	ND	ND	0.068%	ND	0.732%	0.002%	ND	ND
SP8	Spain	ND	ND	0.132%	ND	7.457%	0.003%	ND	ND
SP9	Spain	ND	ND	0.287%	ND	5.294%	0.072%	ND	0.002%
SP10	Spain	ND	ND	0.317%	ND	9.172%	ND	ND	2.051%
SP11	Spain	ND	0.003%	0.713%	ND	0.199%	ND	ND	0.334%
SP12	Spain	ND	ND	2.780%	ND	0.106%	ND	ND	0.004%
UK1	UK	ND	0.001%	2.010%	ND	0.780%	4.119%	0.065%	0.691%
UK2	UK	ND	ND	1.576%	ND	0.073%	0.153%	0.025%	2.474%
UK3	UK	ND	0.009%	1.652%	ND	0.651%	26.158%	0.002%	10.828%
UK4	UK	ND	ND	3.710%	ND	0.314%	2.510%	0.160%	12.150%
UK5	UK	ND	ND	1.852%	ND	0.077%	9.449%	0.025%	4.456%
UK6	UK	ND	ND	1.817%	ND	0.541%	4.146%	0.026%	4.600%
UK7	UK	ND	0.001%	1.250%	ND	6.684%	4.094%	1.302%	1.239%

**Sample**	**Origin**	*F. redolens*		FSSC		FTSC		*F. vert./F. subgl.*	
		SOIL	GRAIN	SOIL	GRAIN	SOIL	GRAIN	SOIL	GRAIN
**SP1**	Spain	0.108%	ND	ND	ND	5.915%	0.265%	0.007%	ND
SP2	Spain	0.204%	ND	ND	ND	0.731%	0.003%	0.014%	ND
SP3	Spain	0.110%	ND	0.004%	ND	4.356%	0.008%	0.007%	0.010%
SP4	Spain	0.216%	0.002%	ND	ND	1.955%	0.009%	0.007%	ND
SP5	Spain	0.058%	ND	0.002%	ND	0.499%	0.029%	0.010%	0.001%
SP6	Spain	0.194%	ND	ND	ND	3.797%	0.008%	0.045%	ND
SP7	Spain	0.662%	ND	ND	ND	2.878%	ND	0.001%	ND
SP8	Spain	0.196%	ND	0.019%	ND	0.123%	ND	0.046%	ND
SP9	Spain	1.205%	ND	0.004%	ND	0.835%	ND	0.105%	0.001%
SP10	Spain	0.956%	ND	ND	ND	0.681%	ND	0.046%	ND
SP11	Spain	0.293%	ND	ND	ND	8.347%	0.011%	0.007%	ND
SP12	Spain	1.035%	0.002%	0.257%	ND	0.642%	0.007%	0.006%	0.003%
UK1	UK	0.861%	ND	0.196%	ND	0.698%	0.042%	0.003%	ND
UK2	UK	0.430%	ND	0.943%	ND	0.067%	0.119%	ND	0.001%
UK3	UK	1.356%	ND	0.114%	ND	0.171%	0.596%	0.014%	0.001%
UK4	UK	0.150%	ND	0.509%	ND	1.262%	2.031%	0.001%	ND
UK5	UK	0.219%	ND	0.100%	ND	2.231%	26.895%	ND	0.018%
UK6	UK	0.332%	ND	0.035%	ND	0.497%	45.962%	0.004%	ND
UK7	UK	2.754%	ND	0.188%	ND	0.685%	0.468%	0.021%	ND

ND: Not detected. Only the OTU which were present in two or more samples and with a count higher than 10 in at least one sample are represented.
